# Factors Associated With Dysfunction of Glymphatic System in Patients With Glioma

**DOI:** 10.3389/fonc.2021.744318

**Published:** 2021-09-23

**Authors:** Cheng Hong Toh, Tiing Yee Siow

**Affiliations:** ^1^ Department of Medical Imaging and Intervention, Chang Gung Memorial Hospital at Linkou, Tao-Yuan, Taiwan; ^2^ Chang Gung University College of Medicine, Tao-Yuan, Taiwan

**Keywords:** glioma, glymphatic system, peritumoral brain edema, analysis along perivascular space (ALPS) index, diffusion tensor imaging

## Abstract

**Objectives:**

Rodent experiments have provided some insights into the changes of glymphatic function associated with glioma growth. The diffusion tensor image analysis along the perivascular space (DTI-ALPS) method offers an opportunity for the noninvasive investigation of the glymphatic system in patients with glioma. We aimed to investigate the factors associated with glymphatic function changes in patients with glioma.

**Materials and Methods:**

A total of 201 glioma patients (mean age = 47.4 years, 116 men; 86 grade II, 52 grade III, and 63 grade IV) who had preoperative diffusion tensor imaging for calculation of the ALPS index were retrospectively included. Information collected from each patient included sex, age, tumor grade, *isocitrate dehydrogenase 1* (*IDH1*) mutation status, peritumoral brain edema volume, tumor volume, and ALPS index. Group differences in the ALPS index according to sex, tumor grade, and *IDH1* mutation status were assessed using analysis of covariance with age adjustment. Linear regression analyses were performed to identify the factors associated with the ALPS index.

**Results:**

Group comparisons revealed that the ALPS index of grade II/III gliomas was significantly higher than that of grade IV gliomas (*p* < 0.001). The ALPS index of *IDH1* mutant gliomas was significantly higher than that of *IDH1* wild-type gliomas (*p* < 0.001). On multivariable linear regression analysis, *IDH1* mutation (*β* = 0.308, *p* < 0.001) and peritumoral brain edema volume (*β* = −0.353, *p* < 0.001) were the two independent factors associated with the ALPS index.

**Conclusion:**

*IDH1* wild-type gliomas and gliomas with larger peritumoral brain edema volumes were associated with a lower ALPS index, which may reflect impaired glymphatic function.

## Introduction

The glymphatic system has been recently recognized as a pathway for waste clearance and maintaining fluid balance in the brain parenchymal interstitium (1). Cerebrospinal fluid (CSF) from the subarachnoid space flows into the brain parenchyma through periarterial spaces of the penetrating arteries and, under the influence of aquaporin-4 (AQP4) water channels, mixes with parenchymal interstitial fluid. The interstitial fluid and its solutes then move into the perivenous and perineuronal spaces, thereafter leaving the brain parenchyma. The discovery of the glymphatic system led to a new perspective into the pathogenesis of brain diseases such as neurodegeneration (2) and acute ischemic infarct (3). Recent studies have shown that the glymphatic system may play an essential role in brain tumor immunity and might be targeted in brain tumor immunotherapy (4).

Rodent experiments have provided some insights into the changes of glymphatic function associated with glioma growth. In rodent studies, reduced CSF efflux rate (5) and remodeling of the glymphatic pathway (4) were observed in glioma-bearing mice, and these changes may be associated with the formation of peritumoral brain edema. Despite substantial knowledge having been gained from animal studies, further research is necessary to confirm whether the findings regarding the glymphatic system of animals apply to humans. Noninvasive approaches such as the morphological assessment of intracranial perivascular spaces with structural magnetic resonance imaging (MRI) (6–9) and measurement of water diffusivity using diffusion MRI (10–12) have been proposed for the evaluation of human glymphatic function.

The analysis along the perivascular space (ALPS) index (10) is a diffusion metric derived from diffusion tensor imaging (DTI). It is thought to reflect the diffusivity along the perivascular spaces of medullary veins at the level of the lateral ventricle body and, thus, may serve as an estimate of human glymphatic function. Alterations of the ALPS index correlate with the Mini-Mental State Examination scores in patients with Alzheimer’s disease and are thought to reflect glymphatic dysfunction. The ALPS index is also significantly lower in normal pressure hydrocephalus patients and points toward glymphatic dysfunction, as indicated by delayed clearance of intrathecally injected gadobutrol (13, 14).

The ALPS index offers an opportunity for the noninvasive investigation of the human glymphatic system. Thus, we took advantage of this method to evaluate the glymphatic system in patients with glioma. We aimed to investigate the correlations of glymphatic function with the volumes of tumor and peritumoral brain edema, tumor grades, and *isocitrate dehydrogenase 1* (*IDH1*) mutation status.

## Materials and Methods

### Study Subjects

Approval for reviewing patients’ clinical data and preoperative MRI studies was obtained from our Institutional Review Board. Between 2008 and 2018, a total of 239 consecutive patients with a histopathological diagnosis of grade II, III, or IV glioma underwent preoperative DTI at our institution. A total of 38 patients were excluded due to age younger than 18 years (*n* = 2), motion artifacts (*n* = 3), tumors limited to the infratentorial compartment (*n* = 4), and the *IDH1* mutation status not being available (*n* = 29). Thus, a total of 201 patients (85 women and 116 men, mean age = 47.4 ± 15.5 years, range = 18–91 years) were analyzed. None of the patients had begun corticosteroid treatment, radiation therapy, chemotherapy, or had previous brain surgery at the time of their MRI studies.

### Clinical and Imaging Information

Patients’ medical records and MRI studies were retrospectively reviewed to collect information including sex, age, tumor grade, and tumor *IDH1* mutation status. Histopathological diagnosis was made by a board-certified neuropathologist with 20 years of experience according to the 2007 WHO classification of central nervous system tumors before 2016 and thereafter based on the 2016 WHO classification.

### MRI

All MRI studies were performed using a 3-T unit (Magnetom Tim Trio, Siemens, Erlangen, Germany) with a 12-channel phased-array head coil. All examinations included T2-weighted, susceptibility-weighted, DTI, and T1-weighted sequences acquired in the transverse plane before and after administration of 0.1 mmol/kg body weight gadopentetate dimeglumine (Magnevist; Schering, Berlin, Germany).

DTI was performed using a single-shot echo planar sequence (EPI) with the following parameters: repetition time/echo time (TR/TE), 5,800/83 ms; diffusion gradient encoding in 20 directions; *b* = 0, 1,000 s/mm^2^; field of view (FOV), 256 × 256 mm; matrix size, 128 × 128; section thickness, 2 mm; and number of signals acquired, 4. A total of 50–60 sections without intersection gaps were used to cover the cerebral hemispheres, brainstem, and the cerebellum. Generalized autocalibrating partially parallel acquisitions (GRAPPA) with a reduction factor set at 2 were used during DTI acquisitions. Contrast-enhanced T1-weighted images [TR/TE = 2,000/2.63 ms, section thickness = 1 mm, inversion time (TI) = 900 ms, acquisition matrix = 224 × 256, and FOV = 224 × 256 mm] were acquired after completion of the DTI sequence.

### Image Post-Processing and Analysis

The software nordicICE (nordic Image Control and Evaluation, version 2; Nordic Imaging Lab, Bergen, Norway) was used for all volume measurements. All images of each patient were co-registered based on a 3D non-rigid transformation and mutual information. The adequacy of registration was visually assessed and manual adjustments were performed by changing the transformation parameters of translation, rotation, and/or scaling, as necessary. The ALPS index was measured with 3D Slicer, version 4.10.2 (http://www.slicer.org). Blinded to histopathologic diagnoses, two neuroradiologists (with 16 and 6 years of experience) independently performed all measurements. If the tumors were found in both hemispheres, only those in the hemisphere with the larger tumor were selected for measurements of volumes. If multiple tumors or peritumoral brain edema areas were present, all were included as long as their sizes were larger than 1 × 1 cm^2^.

### Measurements of Volumes of Peritumoral Brain Edema, Whole Tumor, Enhancing Tumor Portions, Non-Enhancing Tumor Portions, Necrotic Portions, and Hemorrhagic Portions

**Figure 1:**
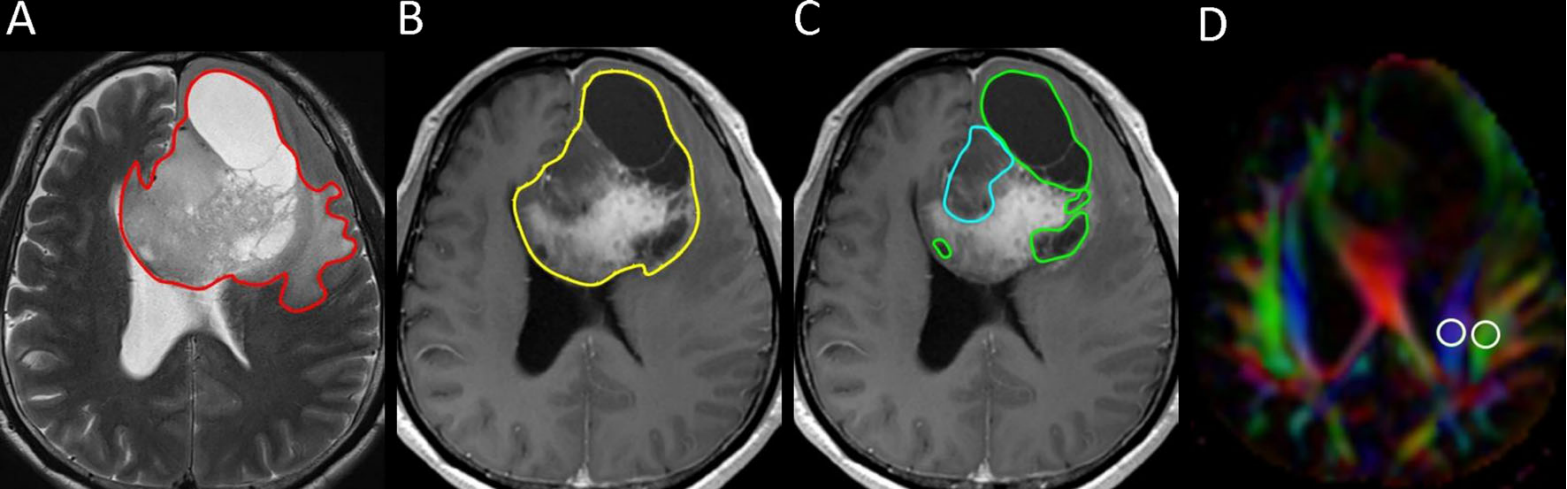
Example of how regions of interest (ROIs) were segmented in a left frontal grade II glioma. **(A)** Transverse T2-weigthed image showing a manually drawn polygonal ROI (*red*) that includes the entire peritumoral brain edema and the whole tumor. **(B, C)** On contrast-enhanced T1-weighted images, the ROIs of whole tumor (*yellow*), non-enhancing tumor (*blue*), and necrotic portion (*green*) are shown. **(D)** Directionally encoded color map illustrating the ROIs of the projection (*blue area*) and association (*green area*) fibers in the left periventricular region for calculation of the analysis along the perivascular space (ALPS) index.

One polygonal region of interest (ROI) was first placed on each T2-weighted image to include the entire peritumoral brain edema and tumor, followed by another ROI drawn to include the entire tumor on each contrast-enhanced T1-weighted image. Subtracting the second ROI from the first ROI yielded the isolated peritumoral brain edema area. If non-enhancing, necrotic, and hemorrhagic tumor portions were present, they were measured by placing the ROIs on T2-weighted or contrast-enhanced T1-weighted images with reference to other pulse sequence images. In all the slices covering the whole tumor, the ROIs were drawn. Non-enhancing tumor portions were tumor components that showed T2-weighted/fluid-attenuated inversion recover (FLAIR) hyperintensity with corresponding T1-weighted hypointensity and no contrast enhancement. Necrotic tumor portions were tumor components that demonstrated central T2-weighted hyperintensity (i.e., fluid) with surrounding contrast enhancement. Hemorrhagic tumor portions were tumor components that had T1-weighted hyperintensity, T2-weighted hypointensity, or susceptibility-weighted hypointensity. Subtracting the ROIs of the non-enhancing, necrotic, and hemorrhagic tumor portions from the tumor ROI yielded the enhancing tumor area. The slice volume of each ROI was computed by multiplying the area by slice distance (slice thickness + slice gap). The total volumes of the peritumoral brain edema, whole tumor, enhancing tumor portion, non-enhancing tumor portion, necrotic portion, and hemorrhagic portion were calculated by summing up all the slice volumes. An example of ROI segmentation is shown in **Figure 1**.

### Measurement of the ALPS Index

The diffusion tensor image analysis along the perivascular space (DTI-ALPS) method (10) was used to evaluate the glymphatic function. This method evaluates the diffusivity along with the perivascular space on a transverse slice at the level of the lateral ventricle body. The medullary veins, accompanied by their perivascular spaces, run perpendicular to the ventricular walls at the level of the lateral ventricular bodies in a right–left or a left–right direction (i.e., *x*-axis in the image coordinate). In this level, the corticofugal corona radiata projection fibers run in the craniocaudal direction (i.e., *z*-axis in the image coordinate) adjacent to the lateral ventricles. The superior longitudinal fascicle, which represents the association fibers, runs in the anterior–posterior direction (i.e., *y*-axis in the image coordinate) and is located lateral to the corona radiata. As the perivascular space is nearly perpendicular to both the projection fibers and association fibers, the mean *x*-axis diffusivity in both fibers (*D*_xproj_ and *D*_xassoc_ for *x*-axis diffusivity in the projection fiber and the association fiber, respectively) can reflect the change in perivascular flow, after being normalized to the mean diffusivity that is perpendicular to the *x*-axis and to the direction of the fiber tracts (*y*-axis for the projection fiber, where the diffusivity is denoted as *D*_yproj_; *z*-axis for the association fiber, where the diffusivity is denoted as *D*_zassoc_). To estimate the glymphatic activity, the ALPS index is defined as follows:


(1) $$\text{ALPS index}=\frac{\text{mean}(D_{\text{xproj}},D_{\text{xassoc}})}{\text{mean}(D_{\text{yproj}},D_{\text{zassoc}})}$$

Diffusion metric images were generated by using 3D Slicer, version 4.10.2 (http://www.slicer.org). The diffusion tensor is calculated based on the “Diffusion Tensor Estimated function” in 3D Slicer. D*xx*, D*yy*, and D*zz* were extracted from the tensor data using a self-written Python script in the 3D Slicer Python interactor. The ROIs of the projection (mean size = 35 ± 19 mm^2^) and association fibers (mean size = 30 ± 18 mm^2^) ipsilateral to the tumors were drawn on a slice at the level of the lateral ventricular body based on a directionality encoded map. The ALPS index was computed according to Equation 1 above. An example of ROI placement for ALPS index measurement is shown in **Figure 1**.

### Statistical Analysis

A commercially available statistical software package (SPSS 22; IBM, Armonk, NY, USA) was used for analysis, and *p*-values <0.05 were considered to indicate a statistical significance. Continuous variables are denoted as the mean ± standard deviation, unless otherwise noted. The Kolmogorov–Smirnov test was used to assess the normality of continuous variables. Variance inflation factors were used to detect multicollinearity. Interobserver variability in the measurements of volumes and the ALPS index was assessed by intraclass correlation coefficients (ICCs) with 95% confidence intervals (CIs) based on an absolute‐agreement, two‐way random‐effects model. The final values of all measurements were obtained by taking the mean of the two observers’ independent measurements.

Group differences in the ALPS index according to sex (man *vs*. woman), tumor grade (grade II *vs*. III *vs*. IV), and *IDH1* mutation status (mutant *vs*. wild-type) were assessed using analysis of covariance with age adjustment (15, 16). Additional Bonferroni correction was performed for tumor grade.

The associations of ALPS index with age, sex, histological grade, *IDH1* mutation status, whole tumor volume, enhancing tumor volume, non-enhancing tumor volume, necrotic portion volume, and hemorrhagic portion volume were first analyzed with univariable linear regression. All variables were entered as potential covariates in the stepwise multivariable linear regression analysis to identify independent factors associated with the ALPS index.

## Results

**Table 1** summarizes the patients’ characteristics and all measurements. Among the 201 patients with gliomas, 86 had grade II, 52 had grade III, and 63 had grade IV. A total of 107 (53.2%) tumors were *IDH1*-mutant. Eighty-four (41.8%) patients had glioma involving the right cerebral hemisphere.

**Table 1:** Patient characteristics and all measurements.

Characteristics	Grade II	Grade III	Grade IV	All
No. of patients	86	52	63	201
Mean age ± SD (years)	41.4 ± 13.3	45.2 ± 13.6	57.5 ± 15.1	47.4 ± 15.5
Sex				
Woman	41	23	21	85
Man	45	29	42	116
Tumor location				
Right cerebral	37	16	31	84
Left cerebral	49	36	32	117
*IDH1* mutation				
Wild-type	20	18	56	94
Mutant	66	34	7	107
Peritumoral edema volume (cm^3^)	13.81 ± 18.36	17.04 ± 21.11	50.33 ± 35.70	26.09 ± 30.38
Whole tumor volume (cm^3^)	52.82 ± 45.56	58.69 ± 40.92	50.04 ± 27.35	53.47 ± 39.40
Enhancing tumor volume (cm^3^)	1.59 ± 6.21	12.27 ± 19.26	31.22 ± 15.46	16.34 ± 18.58
Non-enhancing tumor volume (cm^3^)	47.02 ± 42.57	33.11 ± 30.57	3.69 ± 18.75	29.84 ± 38.25
Necrotic portion volume (cm^3^)	1.94 ± 7.30	9.34 ± 22.39	8.10 ± 13.05	5.78 ± 14.65
Hemorrhagic portion volume (cm^3^)	1.18 ± 10.95	3.05 ± 11.43	8.06 ± 12.55	3.82 ± 11.91
ALPS index	1.454 ± 0.186	1.421 ± 0.214	1.220 ± 0.112	1.372 ± 0.202

Except where indicated, data are numbers of patients.

SD, standard deviation; ALPS, analysis along the perivascular space.

There were excellent interobserver agreements (ICC = 0.822–0.948, *p* < 0.001) in the measurements of peritumoral brain edema volumes, whole tumor volumes, enhancing tumor volumes, non-enhancing tumor volumes, necrotic portions volumes, hemorrhagic portions volumes, and the ALPS index. The mean volumes (in cubic centimeters) of the peritumoral brain edema, whole tumor, enhancing tumor, non-enhancing tumor, necrotic portion, and hemorrhagic portion were 26.09 ± 30.38, 53.47 ± 39.40, 16.34 ± 18.58, 29.84 ± 38.25, 5.78 ± 14.65, and 3.82 ± 11.91, respectively. The mean ALPS index for grade II, grade III, and grade IV gliomas were 1.454 ± 0.186, 1.421 ± 0.214, and 1.220 ± 0.112, respectively.

**Figure 2:**
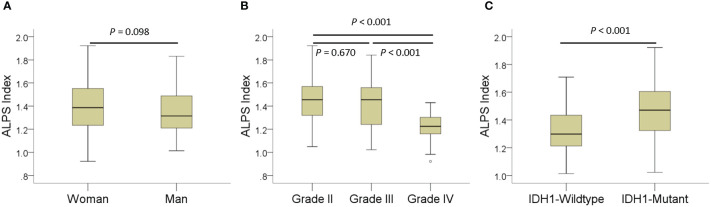
Boxplots showing differences in the analysis along the perivascular space (ALPS) index according to sex **(A)**, tumor grade **(B)**, and *IDH1* mutation status **(C)**.

The results of ALPS index comparisons according to sex, tumor grade, and *IDH1* mutation status with age adjustment are summarized in **Table 2**. The ALPS index of women was not significantly different from that of men (*p* = 0.098). The ALPS index of grade II and grade III gliomas was significantly higher than that of grade IV gliomas (*p* < 0.001). However, the ALPS index was not different between grade II and III gliomas (*p* = 0.670). The ALPS index of *IDH1* wild-type gliomas was significantly lower than that of *IDH1* mutant gliomas (*p* < 0.001). **Figure 2** shows the differences in the ALPS index according to sex, tumor grade, and *IDH1* mutation status.

**Table 2:** Comparisons of the ALPS index according to sex, tumor grade, and *IDH1* mutation status with age adjustment.

Characteristics	ALPS index	Pairwise comparison	*p*-value	95% CI
Sex		Woman *vs*. man	0.098	−0.009 to 0.105
Woman	1.405 ± 0.216			
Man	1.348 ± 0.190			
Tumor grade		Grade II *vs*. III	0.670	−0.037 to 0.112
Grade II	1.454 ± 0.186	Grade II *vs*. IV	<0.001	0.178–0.333
Grade III	1.421 ± 0.214	Grade III *vs*. IV	<0.001	0.135–0.301
Grade IV	1.220 ± 0.112			
*IDH1* mutation		Wild-type *vs*. mutant	<0.001	−0.235 to −0.097
Wild-type	1.311 ± 0.151			
Mutant	1.466 ± 0.201			

Data are the mean ± SD.

CI, confidence interval; ALPS, analysis along the perivascular space.

**Figure 3:**
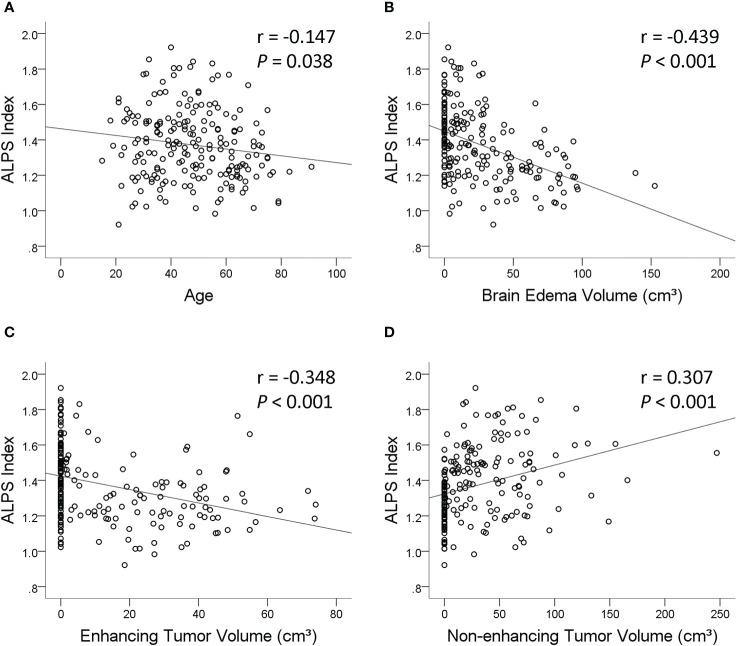
Scatterplots with regression line showing the correlations of the analysis along the perivascular space (ALPS) index with age **(A)**, peritumoral brain edema volume **(B)**, enhancing tumor volume **(C)**, and non-enhancing tumor volume **(D)**.

The results of linear regression analyses of the factors associated with the ALPS index are summarized in **Table 3**. On univariable linear regression analysis, the ALPS index correlated with age (*β* = −0.147, *p* = 0.038), sex (*β* = −0.139, *p* = 0.049), tumor grade (*β* = −0.361, *p* < 0.001), *IDH1* mutation (*β* = 0.384, *p* < 0.001), peritumoral brain edema volume (*β* = −0.439, *p* < 0.001), enhancing tumor volume (*β* = −0.348, *p* < 0.001), and non-enhancing tumor volume (*β* = 0.307, *p* < 0.001). The associations of the ALPS index with whole tumor volume (*β* = 0.101, *p* = 0.155), necrotic portion volume (*β* = −0.058, *p* = 0.410), and hemorrhagic portion volume (*β* = 0.002, *p* = 0.983) were not statistically significant. **Figure 3** illustrates the correlations of the ALPS index with age, peritumoral brain edema volume, enhancing tumor volume, and non-enhancing tumor volume.

**Table 3:** Univariable and multivariable linear regression analyses of factors associated with the ALPS index.

Characteristics	ALPS Index			
	Univariate linear regression		Multivariate linear regression	
	*β*	*p-*value	*β*	*p-*value
Age (years)	−0.147	0.038	0.075	0.351
Sex	−0.139	0.049	0.001	0.993
Tumor grade	−0.361	<0.001	−0.127	0.149
*IDH1* mutation	0.384	<0.001	0.308	<0.001
Brain edema volume	−0.439	<0.001	−0.353	<0.001
Whole tumor volume	0.101	0.155	0.119	0.122
Enhancing tumor volume	−0.348	<0.001	0.024	0.778
Non-enhancing tumor volume	0.307	<0.001	0.108	0.187
Necrotic portion volume	−0.058	0.410	−0.033	0.653
Hemorrhagic portion volume	0.064	0.454	0.133	0.092

ALPS, analysis along the perivascular space; β, standardized coefficient.

On stepwise multivariable linear regression analysis, *IDH1* mutation (*β* = 0.308; *p* < 0.001) and peritumoral brain edema volume (*β* = −0.353; *p* < 0.001) were the two independent factors associated with the ALPS index. A lower ALPS index was associated with *IDH1* wild-type gliomas and larger peritumoral brain edema volume.

## Discussion

In the present study, the *IDH1* mutation status of gliomas and the peritumoral brain edema volume were two independent factors associated with the ALPS index. As reflected by a lower ALPS index, glymphatic function was impaired in *IDH1* wild-type gliomas and gliomas associated with larger peritumoral brain edema volumes. To our knowledge, this is the first study of human glymphatic function in patients with glioma.

The inverse correlation between the ALPS index and peritumoral brain edema volume was observed in meningiomas (17). It was proposed that tumor growth disrupts the balance between periarterial CSF influx and perivenous interstitial fluid efflux and results in the accumulation of interstitial fluid, i.e., peritumoral brain edema. A higher glymphatic function may facilitate interstitial fluid clearance and reduce or even prevent edema. In contrast, insufficient glymphatic function for interstitial fluid clearance may contribute to edema formation. Therefore, glymphatic dysfunction may explain the pathogenesis of peritumoral brain edema in meningiomas, which are extra-axial and have no direct contact with the brain interstitium.

The pathogenesis of peritumoral brain edema in gliomas is traditionally thought to represent the net transport of fluid from the intravascular compartment into the brain interstitium due to the proliferation of microvessels that have defects in their inter-endothelial tight junctions (18). However, this theory does not explain the formation of peritumoral brain edema in low-grade gliomas with intact tight junctions (19, 20). In our study, we also observed an inverse relationship between the ALPS index and peritumoral brain edema volume. This finding suggests that the peritumoral brain edema associated with intra-axial tumors (i.e., gliomas) may also be related to glymphatic dysfunction.

A recent study has shown that changes in the supporting structures of the blood–brain barrier, such as astrocytes, pericytes, and microglial cells, may also be associated with the influx of fluid into the brain interstitium (21). The astrocyte covering of brain microvessels seems to be rate limiting to water movement (21), and AQP4 water channels located on astrocytic foot processes may play a significant role in peritumoral brain edema formation. A strong correlation between peritumoral brain edema and upregulated astrocyte AQP4 expression in human gliomas suggests that increased AQP4 expression may be essential to the pathogenesis of peritumoral brain edema (22). Since AQP4 water channels are part of the glymphatic system, we speculate that there may an association between the ALPS index and the expression of AQP4 water channels. Further studies are needed to establish the role of the ALPS index as an imaging marker of AQP4 expression in gliomas.

The changes of glymphatic function associated with glioma growth have only been investigated in a few animal studies. In a study using an orthotopic xenograft glioma model (5), glioma growth caused glymphatic dysfunction with reduced CSF flow to the extracranial space. In our study, the glymphatic function of *IDH1* wild-type gliomas, measured with the ALPS index, was significantly lower than that of *IDH1* mutant gliomas. To the best of our knowledge, no human studies have reported the association between glymphatic function and the *IDH1* mutation status of gliomas.

*IDH1* wild-type gliomas are known to behave more aggressively than *IDH1* mutant gliomas (23). We speculate that the spread of *IDH1* wild-type gliomas is associated with greater disruption to the flow of interstitial fluid and, consequently, lower glymphatic function. Alternatively, tumor-associated glymphatic pathway remodeling may be responsible for the differences in glymphatic function between *IDH1* wild-type and *IDH1* mutant gliomas. In mice harboring gliomas, glymphatic function is increased for waste and fluid clearance by extensive growth of meningeal lymphatic vessels, which are downstream of the glymphatic pathway (4). A previous study showed that the growth of glioblastomas (mostly *IDH1* wild-type) from small to large tumors could take as short as 1.5 months (24). In contrast, *IDH1* mutant gliomas are known for longer survival and may be stable for years (25). We therefore speculate that the lower glymphatic function of *IDH1* wild-type gliomas may be related to their short duration of tumor growth, which does not allow extensive remodeling of the glymphatic pathway.

Although the mechanism for the association between glymphatic function and *IDH1* mutation status is unclear, the relationship has revealed some potential utilities for the ALPS index. Many MR imaging markers have been found to be useful in predicting the *IDH1* mutation status in gliomas (26–29). However, most of them are measured in the tumors, but may not be feasible when a large hemorrhage or necrosis is present. In contrast, the ALPS index is measured in the periventricular region outside of the tumor and, thus, may serve as an alternative marker for *IDH1* mutation prediction. Besides, the ALPS index may also be a prognostic factor for patient survival due to its correlation with *IDH1* mutation status. Further studies are needed to establish the predictive and prognostic roles of the ALPS index.

The anatomical relationship of the perivascular spaces, projection fibers, and association fibers in the periventricular region is the theoretical basis of the DTI-ALPS method. Although this method is based on a deductive model, it is frequently used to investigate human glymphatic function. Its potential to serve as an imaging marker of glymphatic activity has been reported in many clinical conditions such as Alzheimer’s disease (10, 30), normal pressure hydrocephalus (31, 32), Parkinson disease (33, 34), age-related iron deposition (15), diabetic cognitive impairment (35), and meningioma-associated brain edema (17). However, there are limitations to this method and, consequently, our study. Firstly, the diffusion signal measured in clinical settings reflects overall changes in water mobility associated with many processes occurring at scales much smaller than typical MRI voxels. Therefore, we cannot definitely state that the ALPS index is a measure of glymphatic function. Secondly, the ALPS index could only measure the perivascular water flow that runs in a right-to-left direction and in the periventricular region. Further studies are needed to establish its correlation with overall glymphatic function. Finally, our study is a snapshot in time and does not include longitudinal data on temporal changes of the ALPS index and peritumoral brain edema volume following treatment. These pieces of information would be helpful to further establish the role of the glymphatic system in brain edema formation.

## Conclusions

In conclusion, *IDH1* wild-type gliomas and gliomas with larger peritumoral brain edema volumes were associated with a lower ALPS index, which may reflect an impaired glymphatic function. The lower ALPS index in *IDH1* wild-type gliomas may suggest an association between glymphatic dysfunction and tumor aggressiveness. In addition, the correlation between larger peritumoral brain edema volumes and a lower ALPS index suggests that the formation of peritumoral brain edema may be related to glymphatic dysfunction.

## Data Availability Statement

The raw data supporting the conclusions of this article will be made available by the authors, without undue reservation.

## Ethics Statement

The studies involving human participants were reviewed and approved by Chang Gung Medical Foundation Institutional Review Board. Written informed consent for participation was not required for this study in accordance with the national legislation and the institutional requirements.

## Author Contributions

CT and TS contributed to the conception and design of the study and to the acquisition and analysis of data. CT drafted the text, prepared the figures, and wrote the first draft of the manuscript. All authors read the final manuscript. All authors contributed to the article and approved the submitted version.

## Funding

This study was funded by the National Science Council, Taiwan (NSC98-2314-B-182A-051-MY3 to CT).

## Conflict of Interest

The authors declare that the research was conducted in the absence of any commercial or financial relationships that could be construed as a potential conflict of interest.

## Publisher’s Note

All claims expressed in this article are solely those of the authors and do not necessarily represent those of their affiliated organizations, or those of the publisher, the editors and the reviewers. Any product that may be evaluated in this article, or claim that may be made by its manufacturer, is not guaranteed or endorsed by the publisher.
